# Non-invasive *in vivo* imaging of brain and retinal microglia in neurodegenerative diseases

**DOI:** 10.3389/fncel.2024.1355557

**Published:** 2024-01-29

**Authors:** Fazeleh Etebar, Damien G. Harkin, Anthony R. White, Samantha J. Dando

**Affiliations:** ^1^Centre for Immunology and Infection Control, School of Biomedical Sciences, Faculty of Health, Queensland University of Technology (QUT), Brisbane, QLD, Australia; ^2^Mental Health and Neuroscience Program, QIMR Berghofer Medical Research Institute, Herston, QLD, Australia; ^3^Centre for Vision and Eye Research, School of Biomedical Sciences, Faculty of Health, Queensland University of Technology (QUT), Brisbane, QLD, Australia

**Keywords:** microglia, non-invasive *in vivo* imaging, positron emission tomography, optical coherence tomography, confocal scanning laser ophthalmoscopy, adaptive optics, Alzheimer’s disease, multiple sclerosis

## Abstract

Microglia play crucial roles in immune responses and contribute to fundamental biological processes within the central nervous system (CNS). In neurodegenerative diseases, microglia undergo functional changes and can have both protective and pathogenic roles. Microglia in the retina, as an extension of the CNS, have also been shown to be affected in many neurological diseases. While our understanding of how microglia contribute to pathological conditions is incomplete, non-invasive *in vivo* imaging of brain and retinal microglia in living subjects could provide valuable insights into their role in the neurodegenerative diseases and open new avenues for diagnostic biomarkers. This mini-review provides an overview of the current brain and retinal imaging tools for studying microglia *in vivo*. We focus on microglia targets, the advantages and limitations of *in vivo* microglia imaging approaches, and applications for evaluating the pathogenesis of neurological conditions, such as Alzheimer’s disease and multiple sclerosis.

## Introduction

The central nervous system (CNS) parenchyma is populated with resident macrophages called microglia, which contribute to regulation of neurodevelopment, CNS homeostasis, inflammation and injury repair (Michell-Robinson et al., 2015; McMenamin et al., 2019). Microglia are implicated in the pathogenesis of several neurodegenerative conditions, including Alzheimer’s disease (AD) (Cherry et al., 2015; Shi et al., 2019), multiple sclerosis (MS) (Voet et al., 2019) and Parkinson’s disease (Guo et al., 2020), with recent studies suggesting that different microglia subtypes with varying functional responses may be involved in CNS diseases (Olah et al., 2011; Flowers et al., 2017; Keren-Shaul et al., 2017; Hammond et al., 2019; Masuda et al., 2020).

Much of our understanding of microglia in humans and animal models comes from studies of fixed or *ex vivo* tissue, *in vitro* cell cultures or ‘omics’ analysis of microglia isolated from CNS tissue. However, tissue processing methods may artificially shift microglia into various reactive states that are not representative of their *in vivo* status (Marsh et al., 2022). Approaches to non-invasively study microglia in their physiological environment in living subjects are therefore of interest to advance our understanding of these cells and their involvement in CNS diseases. As ‘first line’ responders in CNS immune defense, non-invasive *in vivo* evaluation of microglia has been proposed as a tool for the diagnosis and monitoring of neuroinflammation in neuropathological conditions (Tucker et al., 2016; Beaino et al., 2017, 2020; Ardaya et al., 2020; Coda et al., 2021). An altered CNS inflammatory state is postulated to occur prior to the onset of pathology in many neurodegenerative conditions (Hansen et al., 2017; Gazestani et al., 2023), suggesting that non-invasive *in vivo* microglia imaging could be used to identify early signs of disease (Tucker et al., 2016; Beaino et al., 2017, 2020; Ardaya et al., 2020; Coda et al., 2021). Furthermore, the ability to monitor microglia in a non-invasive manner may also inform patient treatment, especially considering that these cells are being investigated as immunotherapeutic targets for several neurological and ocular conditions (Liu et al., 2022; Gao et al., 2023).

Here, we review non-invasive techniques that have been used to image microglia in the brain and retina of living subjects, including positron emission tomography (PET), optical coherence tomography (OCT), confocal scanning laser ophthalmoscopy (cSLO) and adaptive optics. We discuss these imaging approaches in the context of AD, MS, and their animal models.

## Non-invasive approaches for *in vivo* microglia imaging

### Positron emission tomography

Positron emission tomography is the most commonly employed non-invasive approach for imaging brain inflammation; used for quantitative assessment of neuroinflammation and longitudinal visualization of CNS immune cells in clinical studies and animal models (Politis et al., 2012b). This technique uses radiolabelled tracers, which comprise a ligand that binds to protein targets, and a positron-emitting isotope that is detected using nuclear medicine. PET radiotracers for imaging of targets in the brain must meet basic requirements, such as the ability to cross the blood-brain barrier, specific binding to the target with high affinity, and metabolic stability (Pike, 2009). A limitation of commercial PET scanners is the relatively low spatial resolution, reported to be 2–6 mm in dedicated brain PET imaging devices (Catana, 2019).

Positron emission tomography targets for imaging neuroinflammation have been reviewed elsewhere (Tronel et al., 2017; Beaino et al., 2021), and their application in neurodegenerative diseases will be covered in greater detail in subsequent sections. The most widely used target for PET imaging of neuroinflammation is Translocator protein 18 kDa (TSPO) (Jain et al., 2020). Although highly expressed by activated microglia, TSPO lacks specificity as it is also expressed by other brain cell types during disease, including astrocytes, endothelial cells and infiltrating immune cells (Kaunzner et al., 2019; Nutma et al., 2019; Gui et al., 2020). Other targets that have been investigated for PET imaging of neuroinflammation include cyclooxygenase (COX) isoforms (Shrestha et al., 2020), cannabinoid receptor type 2 (CB_2_R) (Evens et al., 2012) and sphingosine-1-phosphate receptor 1 (S1PR1) (Liu et al., 2016). Whilst none of these targets are exclusively expressed by microglia, they have been shown to be upregulated in the brain during pathological conditions and therefore can indicate a broad neuroinflammatory state.

The ability to selectively target microglia and their subtypes using PET imaging would be a significant step forward for *in vivo* brain imaging, potentially enabling new insights into the contribution of these cells to the pathogenesis of neurodegenerative diseases. Limited progress toward the goal of microglia-specific PET imaging has been made using radiotracers targeting Purinergic 2Y receptor type 12 (P2RY12). P2RY12 is highly expressed in homeostatic conditions and can distinguish microglia from other brain cells and border-associated macrophages (Sasaki et al., 2003; Beaino et al., 2017; Mildner et al., 2017). The expression of P2RY12 is altered during CNS diseases, with immunohistochemical studies of human brain tissue suggesting that reduced microglial P2RY12 expression occurs in regions of neuropathology and inflammation (Zrzavy et al., 2017; Walker et al., 2020). In contrast, P2RY12 expression may be increased by microglia involved in anti-inflammatory repair processes (Beaino et al., 2017). These properties make P2RY12 an attractive target for PET imaging; however, attempts to develop radiotracers targeting this receptor have largely been unsuccessful to date, demonstrating poor penetration of the blood-brain barrier (Villa et al., 2018; van der Wildt et al., 2021). Further studies to understand the expression of P2RY12 across different neurological diseases and develop radiotracers with improved brain penetration are therefore required.

An alternative marker that has been investigated for PET imaging of brain microglia is colony stimulating factor-1 receptor (CSF-1R) (Horti et al., 2019). Similar to other macrophage populations, microglial development (Ginhoux et al., 2010) and survival (Elmore et al., 2014) are controlled by colony stimulating factor-1 (CSF-1) and its receptor, CSF-1R. In the healthy neural parenchyma, microglia are the sole cells that express CSF-1R, and Horti et al. (2019) developed a CSF-1R targeting PET radiotracer (^11^C-CPPC) that demonstrated high levels of uptake in mice, non-human primates and post-mortem brain tissue of human AD patients. First-in-human use of ^11^C-CPPC revealed promising pharmacokinetic properties and good brain uptake in healthy individuals (Coughlin et al., 2022); whilst these findings were regarded as exciting developments in microglial imaging, an important caveat is that perivascular macrophages and peripheral cells of the monocytic lineage also express CSF-1R (Chitu and Stanley, 2006; Kerkhofs et al., 2020). Therefore, CSF-1R PET imaging of neuroinflammatory conditions involving infiltration of monocytes is unlikely to be truly microglia-specific.

Recent work has focused on Purinergic 2X receptor type 7 (P2RX7) as a promising target for PET imaging of so-called “pro-inflammatory microglia.” *In vitro* studies of primary human microglia polarized into either a pro-inflammatory or anti-inflammatory phenotype demonstrated that P2RX7 is highly expressed by pro-inflammatory (but not anti-inflammatory) microglia (Beaino et al., 2017). Several radiotracers targeting P2RX7 have been evaluated in preclinical and clinical studies, with many showing good pharmacokinetics and brain uptake [reviewed in Beaino et al. (2021)]. A limitation of targeting P2RX7 is that it may also be expressed by astrocytes and oligodendrocytes (albeit at low levels) (Zhao et al., 2021); however, immunostaining of brain tissue with MS active lesions demonstrated that P2RX7 antibodies labeled MHC class II + cells with a microglia-like morphology (Beaino et al., 2017). These findings suggest that P2RX7 is predominantly expressed by microglia.

Overall, combinations of subtype specific markers would be ideal for investigating microglial activation in neurological diseases. Future selection of targets for microglia PET imaging should be guided by the wealth of microglial subtypes (and their markers) that have been identified using transcriptomic approaches in recent years. For example, Keren-Shaul et al. (2017) identified a unique microglia subtype termed ‘disease-associated microglia’ (DAM) in a mouse model of AD and in human brain slices in AD patients. DAM are localized near AD plaques and the transition of homeostatic microglia to DAM begins during early disease (Keren-Shaul et al., 2017). Therefore, non-invasive PET imaging of DAM could be used to detect early disease and monitor progression. The challenge for the field is to identify robust microglia subtype-specific markers that can be used to develop PET radiotracers.

### Retinal imaging techniques

The retina is part of the CNS and enables the visualization and assessment of neurological disease progression through non-invasive imaging (Zhang et al., 2021). Pathological changes occur in the retina in neurodegenerative diseases, and these can be examined using traditional ophthalmic imaging approaches including optical coherence tomography (OCT) and confocal laser scanning ophthalmoscopy (cSLO) (Koronyo et al., 2017; Vij and Arora, 2022; Vujosevic et al., 2023). OCT generates cross-sectional images (typically 4–7 μm axial resolution, 15–20 μm transverse resolution) of the retina by detecting light reflection from the different tissue layers and enables assessment of retinal layer thickness (Bajwa et al., 2015). Techniques such as *en face* OCT and OCT angiography (OCTA) produce transverse retinal images and 3D reconstructions of the retinal vasculature, respectively (Van Velthoven et al., 2006; de Carlo et al., 2015). Confocal scanning laser ophthalmoscopy (cSLO) is used for fundus imaging and offers several modalities, including angiography and retro-illumination. In addition to conventional fundus imaging, cSLO uses lasers with differing wavelengths to produce images of different retinal layers or structural features (Bajwa et al., 2015); however, this technique is limited by a lower axial resolution (∼300 μm) compared to OCT (Mainster et al., 2022). Whilst these techniques enable excellent visualization of the retina for clinical and diagnostic purposes, they are unable to capture detailed information at the cellular and sub-cellular level due to the monochromatic wavefront aberrations of the eye, and therefore studying retinal microglia and their processes in living subjects has been elusive.

To address this challenge, adaptive optics (AO) has been combined with SLO to correct the optical aberrations, enabling fine cellular structures within the retina to be resolved. Geng et al. (2012) developed a custom AO-SLO instrument for non-invasive imaging of the mouse retina and generated the first *in vivo* images of the photoreceptor mosaic in mice. The AO-SLO instrument had a reported axial resolution of ∼10 μm and a submicron transverse resolution, and also enabled individual nerve fiber bundles, blood vessels and capillaries within the mouse retinal nerve fiber layer to be resolved. Furthermore, AO-SLO imaging of transgenic reporter mice enabled visualization of fluorescently labeled ganglion cell bodies, dendrites and axons (Geng et al., 2012). Recent studies have applied this technique to non-invasive imaging of fluorescent microglia in mice (Miller et al., 2019; Joseph et al., 2021). Important advances in near infra-red phase contrast AO-SLO have also enabled label-free imaging of mouse retinal microglia and their process dynamics over time (Joseph et al., 2021). This provides proof-of-concept that phase contrast AO-SLO could be translated to perform *in vivo* microglia imaging in the human eye.

Adaptive optics combined with OCT (AO-OCT) has a resolution of 4.7 μm (axial) and 2.4 μm (lateral) (Liu et al., 2017) and also has potential applications for direct visualization of microglia in the human retina. Several publications have demonstrated that AO-OCT can be used to resolve macrophages (hyalocytes) at the inner limiting membrane (ILM) located at the vitreoretinal interface in the human eye (Liu et al., 2017; Hammer et al., 2020; Kazuhiro et al., 2020). However, ILM macrophages are distinct from microglia, and due to their location exterior to the CNS these cells are not suitable surrogates for studying microglia. To date, AO-OCT studies have been unable to resolve retinal microglia (Hammer et al., 2020), which reside within the outer plexiform layer, inner plexiform layer and ganglion cell layer of the neural retina (McMenamin et al., 2019). Taken together, the recent application of AO to traditional ophthalmic imaging approaches has significantly enhanced retinal imaging capabilities by enabling visualization of cells and cellular structures. Future development in this space will likely lead to non-invasive methods for *in vivo* microglia imaging in the human eye, providing a window into the immune landscape of the CNS. Although, given these are label-free approaches, it is unlikely that they could be adapted to enable targeted imaging of immune cell subtypes in the human retina without the involvement of tracers.

## *In vivo* imaging of brain and retinal microglia in neurodegenerative diseases

### Alzheimer’s disease

Alzheimer’s disease is the most common form of dementia (Gabandé-Rodríguez et al., 2020), characterized by the pathological hallmarks of extracellular deposition of amyloid-β (Aβ) plaques resulting from impairment of Aβ clearance from the CNS (Mawuenyega et al., 2010), and intraneuronal hyperphosphorylated tau protein tangles (Johnson and Stoothoff, 2004). Microglial activation and inflammatory responses are also increasingly recognized as a central feature of AD (Kinney et al., 2018). Microglia undergo a number of functional changes in AD and have beneficial roles, including phagocytosis of Aβ (Gabandé-Rodríguez et al., 2020), lipid metabolism (Claes et al., 2021) and regulation of tau pathology via autophagy (Xu et al., 2021). However, sustained microglial activation and pro-inflammatory signaling can lead to reduced Aβ phagocytosis, exacerbated neuroinflammation and suppression of homeostatic microglia, which contribute to neurodegeneration (Kinney et al., 2018).

### Microglia PET imaging in AD

Positron emission tomography has been extensively used to study neuroinflammation and microglia activation in AD, with a large number of studies reporting that microglial PET target levels are increased in the brains of AD patients (Table 1). TSPO PET in particular has advanced our understanding of the role of microglia in AD, although these findings need to be interpreted carefully due to the non-specificity of TSPO. Increased TSPO levels are positively correlated with Aβ accumulation (Parbo et al., 2017; Dani et al., 2018; Zou et al., 2020) and tau aggregation (Dani et al., 2018) in mild cognitive impairment (MCI) and AD, supporting a role for microglia activation and neuroinflammation in AD. A recent study examined the spatial relationships between microglial activation (determined by TSPO PET), Aβ deposition and tau accumulation in 130 individuals across the spectrum of aging and AD disease progression. This study revealed that microglial activation, potentiated by interactions with Aβ, initiated the spread of tau tangles in the neocortex in a Braak-like pattern (Pascoal et al., 2021). In line with these findings, Rauchmann et al. (2022) reported that microglial activation in AD patients followed a similar spatial distribution to tau along functional connectivity pathways. Taken together, these findings suggest that microglia directly contribute to the pathological hallmarks of AD and highlight the valuable contributions of *in vivo* brain imaging to understanding the pathogenesis of neurodegenerative diseases.

**TABLE 1:** Overview of microglia PET targets and key findings from studies in AD and MS.

PET target	Findings in AD	Findings in MS
TSPO	• Upregulated in human AD and animal models of AD (Zhou et al., 2021). • Increased TSPO PET levels occur in a region-dependent manner in AD (Tournier et al., 2020). • Increased TSPO PET levels are positively correlated with aggregated Aβ and tau in MCI and AD patients (Parbo et al., 2017; Dani et al., 2018; Chandra et al., 2019).	• Diffuse microglial activation observed using TSPO PET in progressive MS (Banati et al., 2000; Politis et al., 2012a; Rissanen et al., 2014; Sucksdorff et al., 2020). • TSPO levels can differentiate chronic active and chronic inactive lesions (Rissanen et al., 2014). • TSPO cannot differentiate different phenotypes of microglia (Nutma et al., 2019). • Increased detection of TSPO predominantly reflects microglia/macrophage density in MS patients, and not activation phenotype (Nutma et al., 2021).
COX1	• COX1-expressing microglia are associated with Aβ plaques in AD (Hoozemans et al., 2001). • COX1 PET levels are increased in the brain in an AD mouse model; COX1 PET tracers may enable tracking of activated microglia associated with Aβ plaque progression (Shukuri et al., 2016).	• Increased COX2 immunoreactivities are observed in activated brain microglia/macrophages in MS (Yiangou et al., 2006).
CB_2_R	• Expressed by neurons, astrocytes and microglia; however, increased levels detected in the brain in human AD and an AD mouse model are predominantly attributed to activated microglia (Benito et al., 2003; Savonenko et al., 2015). • In human AD, a novel CB_2_R PET tracer was detected at significantly lower levels in the brain compared to healthy controls. This may be attributed to loss of CB_2_R expressing neurons in AD (Ahmad et al., 2016).	• Elevated CB2R expression is observed in brain microglia/macrophages in MS (Yiangou et al., 2006).
S1PR1	• Increased levels of S1PR1 were observed in 8- and 14-month-old 5xFAD mice (Jung et al., 2023). • Dysregulation of S1P and S1PR signaling may associate with the development of AD-like pathology (Jung et al., 2023).	• Elevated S1PR1 expression is linked to the activation of glial cells and the infiltration of immune cells (Liu et al., 2016). • The use of MicroPET imaging, employing the radioligand [(11)C]TZ3321, enables the evaluation of S1PR1 expression in the lumbar spinal cord of rats with EAE (Liu et al., 2016). • Evaluation of four 18F-labeled S1PR1 tracers (18F-TZ43113, 18F-TZ35104, 18F-TZ4877, and 18F-TZ4881) in a rat model of multiple sclerosis (MS) revealed that 18F-TZ4877 exhibited the most favorable profile for assessing S1PR1 expression in the EAE rat model of MS (Liu et al., 2020).
P2RX7	• Upregulated by microglia in AD (Francistiová et al., 2020) and modulates chemokine production associated with CD8 + T cell recruitment in Aβ pathology • (Martin et al., 2019)Testing of a novel P2RX7 PET tracer ([11C]SMW139) in human post-mortem brain tissue demonstrated no differences in binding between AD and control tissue (Janssen et al., 2018).	• Increased expression in active MS (Yiangou et al., 2006).
P2RY12	• Downregulated by microglia associated with tau aggregates in human and mouse brain tissue (Maeda et al., 2021).	• PET tracers targeting P2RY12 could be useful in distinguishing the phenotype of microglia in MS (Zrzavy et al., 2017).
CSF-1R	• Depletion of microglia using CSF-1R inhibitors (followed by microglial repopulation) is associated with reduced neuropathology in mouse models of AD (Hu et al., 2021) • CSF-1R PET tracer (^11^C-CPPC) showed elevated brain uptake in a mouse model of AD and post-mortem AD brain tissue compared to controls (Horti et al., 2019).	• Elevated expression in microglia in active MS (Hagan et al., 2020). • CSF-1R PET tracer (^11^C-CPPC) showed elevated brain uptake in EAE mice compared to controls; PET signal intensity was correlated to disease score (Horti et al., 2019).

Interestingly, a longitudinal PET study suggested that microglial activation occurs in two waves during AD disease progression, whereby TSPO signal is initially increased during MCI, then undergoes a longitudinal reduction, followed by a second increase in TSPO signal during AD (Fan et al., 2017). The authors hypothesized that the early peak represents expansion of microglia with a protective phenotype and the later peak represents expansion of pro-inflammatory microglia. However, the ability to study microglia subtypes in living patients remains challenging using existing PET targets. This represents a current limitation of *in vivo* brain imaging, especially considering molecular studies have identified several microglia subtypes with unique functional roles in AD (Kamphuis et al., 2016; Krasemann et al., 2017; Frigerio et al., 2019; Olah et al., 2020; Prater et al., 2021). This includes disease-associated microglia (DAM), which have enhanced phagocytic and lipid metabolism pathways (Keren-Shaul et al., 2017). In mouse models of AD, the switch from a microglial homeostatic phenotype to a disease-associated phenotype involves upregulation of a set of genes, including the AD-associated gene *APOE*, and downregulation of the core microglial transcriptomic signature (Keren-Shaul et al., 2017; Krasemann et al., 2017). The second phase of DAM activation (stage 2 DAM) is mediated by microglial Trem2 (Keren-Shaul et al., 2017). Interestingly, loss-of-function mutations in Trem2 increase the risk of late onset AD, and this may be partially due to the inability of Trem2-deficient microglia to transition to stage 2 DAM (Lewcock et al., 2020). Whist DAM appear to have a neuroprotective role, other microglia subtypes may negatively contribute to neurodegeneration. For example, microglial subtypes enriched in type 1 interferon genes (‘interferon-responsive’ microglia) have been identified in mouse models of AD and in human AD brains (Frigerio et al., 2019; Olah et al., 2020). Roy et al. (2022) demonstrated that microglial type 1 interferon signaling is involved in post-synaptic loss in a model of AD, suggesting a pathogenic role for the interferon-responsive microglia subtype.

The ability to perform non-invasive imaging of functionally distinct microglia subtypes would significantly enhance our understanding of microglial involvement in AD and spatiotemporal changes associated with disease progression. Using PET, this could be achieved with microglia subtype-specific radiotracers. Bartolo et al. (2022) reported an *in silico* approach for identifying microglial candidate genes for PET radiotracer development that could be adapted for this purpose. These authors interrogated published -omics datasets to identify microglia-specific genes that have increased expression in post-mortem AD brain tissue and are associated with neuropathological characteristics (Bartolo et al., 2022). Using this approach, 19 microglia genes were identified and ranked for PET target prioritization. A similar strategy could be employed to determine candidate genes for microglia subtypes, although further studies are first required to obtain a more detailed understanding of microglia subtypes and their transcriptomic signatures in AD.

### Retinal imaging biomarkers and microglia in AD

In recent years there has been significant interest in developing retinal imaging biomarkers for AD. Deposits of Aβ and tau protein have been found in the retina of AD patients, along with other retinal changes including vascular alterations, inflammation and thinning of retinal layers (Ramirez et al., 2017; Snyder et al., 2021; Zhang et al., 2022; Ashraf et al., 2023). Interestingly, in the early stages of disease, preceding Aβ plaque formation in the brain, Aβ plaques were detected in the retina in AD mouse models (Koronyo-Hamaoui et al., 2011; Koronyo et al., 2012), suggesting that retinal imaging may be useful as an early diagnostic tool.

Consistent with observations in the brain suggesting a close spatial relationship between microglia and tau, mouse and human AD studies have shown that retinal tau accumulated in the inner and outer plexiform layers (Chiasseu et al., 2017; den Haan et al., 2018) where microglia are known to be localized. However, unlike brain microglia, retinal microglia have not been widely investigated in AD. Increased microglial density has been reported in the retinae of AD patients compared to controls (Grimaldi et al., 2019; Xu et al., 2022), and it has been proposed that retinal microglia acquire a DAM phenotype during AD based on the expression of a small number of markers (Grimaldi et al., 2019). Studies in mice have also demonstrated changes in retinal microglial phenotypes in AD models, including changes in morphology and spatial distribution (Salobrar-García et al., 2020). Grimaldi et al. (2018) reported that retinal microglia co-localized with Aβ plaques prior to onset of symptoms in 3xTg-AD mice, and that microglia transitioned from a ramified anti-inflammatory phenotype to a pro-inflammatory phenotype as disease progressed. Conversely, in a study of post-mortem donor eyes Xu et al. (2022) demonstrated reduced co-localization of microglia and Aβ in AD retinae compared to control retinae, despite there being an overall increase in retinal microglia immunolabeling in AD. The authors posited that similar to brain microglia, retinal microglia in AD become dysfunctional and have diminished capacity to migrate toward and phagocytose Aβ (Xu et al., 2022).

**FIGURE 1:**
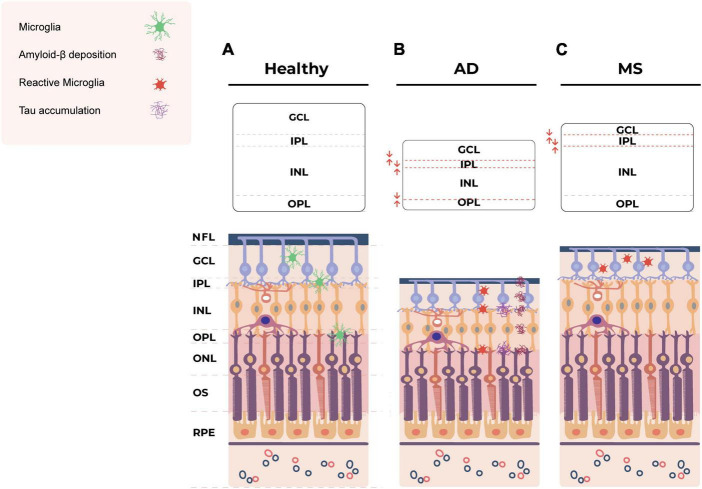
Schematic of the layers of the healthy retina **(A)** and the hypothetical pathological changes in the retina in AD **(B)** and MS **(C)**. **(B)** In AD, Tau accumulation is observed in the inner and outer plexiform layers of the retina (Chiasseu et al., 2017; den Haan et al., 2018). Retinal Aβ plaques are distributed across various layers, including the NFL, GCL, IPL, INL, OPL and even externally to the retina within the sclera (Koronyo-Hamaoui et al., 2011). The eye exhibits structural abnormalities, including decreased thickness of the inner and outer layers (Salobrar-Garcia et al., 2015; Cabrera DeBuc et al., 2019; Jáñez-Escalada et al., 2019; Czakó et al., 2020). Pro-inflammatory, less ramified, neurotoxic microglia are also observed in retina in AD (Zhang et al., 2005; Noailles et al., 2014; Grimaldi et al., 2018; Salobrar-García et al., 2020; Guo et al., 2022). **(C)** In MS, ocular manifestations lead to the thinning of the NFL, GCL and IPL (Cennamo et al., 2016; Choi et al., 2021) and atrophy of the GCL and IPL (Saidha et al., 2015; Petzold et al., 2017). Microglial cell numbers increase in the GCL but decrease in the IPL (Namekata et al., 2019). Amoeboid microglia are present in the inner retinal layers (Jin et al., 2019). AD, Alzheimer’s disease; MS, multiple sclerosis; NFL, nerve fiber layer; GCL, ganglion cell layer; IPL, inner plexiform layer; INL, inner nuclear layer; OPL, outer plexiform layer; ONL, outer nuclear layer; OS, outer segment; RPE, retinal pigment epithelium.

Combined, these studies provide a clear indication of retinal microglial involvement and ocular pathology in AD (summarized in Figure 1). Given the early involvement of the retina in AD, there is a significant need for researchers and clinicians to develop standardized imaging approaches for the assessment of retinal biomarkers, including microglia.

### Multiple sclerosis

Multiple sclerosis, characterized by demyelination and multiple focal lesions, is the most common chronic neurological disease in young adults, affecting 2.8 million people worldwide in 2020 (Walton et al., 2020). MS pathogenesis is thought to be driven by infiltrating autoreactive T cells but also involves a plethora of other infiltrating adaptive and innate immune cell types, as well as resident microglia (Attfield et al., 2022). Strong evidence for microglial involvement in MS was provided by a large genome-wide association study of 47,429 MS and 68,374 control subjects, which revealed that MS susceptibility genes were enriched in microglia but not in other brain cell types (International Multiple Sclerosis Genetics Consortium et al., 2019). Similar findings were reported by Ma et al. (2023), who demonstrated that MS risk genes were significantly enriched in microglial regulatory regions.

Histologically, activated microglia are found in high numbers in active MS lesions and form a rim around mixed active/inactive lesions. Interestingly, microglia are absent in inactive lesions suggesting they play a role in active disease processes (Kuhlmann et al., 2017). Studies of brain tissue from human MS and the experimental autoimmune encephalomyelitis (EAE) animal model have demonstrated that microglia exhibit diverse functions, phenotypes and gene expression profiles in different CNS regions and across different stages of disease (Peferoen et al., 2015; Jordão et al., 2019; Masuda et al., 2019; Schirmer et al., 2019; van der Poel et al., 2019; Miedema et al., 2022). They are thought to contribute to immune-mediated tissue damage during lesion development through various mechanisms including (i) release of reactive oxygen/nitrogen species and toxic levels of glutamate; (ii) sustained pro-inflammatory cytokine production resulting in neuronal and glial dysfunction, and recruitment of infiltrating immune cells; and (iii) antigen presentation to encephalitogenic T cells (Zrzavy et al., 2017; Haimon et al., 2022; Kamma et al., 2022; Montilla et al., 2023). Conversely, microglia also contribute to tissue repair and remyelination in MS by phagocytosing myelin debris, secreting trophic factors, promoting oligodendrocyte maturation, and presenting antigen to regulatory T cells (Lloyd and Miron, 2019; Haimon et al., 2022).

### Microglia PET imaging in MS

In MS, enhanced detection of TSPO in PET imaging studies is correlated with disease severity and clinical disability, indicating that microglial activation/neuroinflammation can be used as a general biomarker of MS disease progression (Banati et al., 2000; Politis et al., 2012a; Rissanen et al., 2014; Sucksdorff et al., 2020). Increased TSPO signal is observed within active lesions, at the rim of mixed active/inactive lesions and has the potential to differentiate chronic active and chronic inactive lesions (Rissanen et al., 2014). Whilst it has been assumed that this is due to increased TSPO expression by activated pro-inflammatory microglia, recent studies of human MS brain tissue have shown that TSPO is expressed in a range of microglia phenotypes and that the increased TSPO signal in lesions predominantly reflects microglia/macrophage density rather than activation status or phenotype (Nutma et al., 2019, 2021).

Elevated levels of the potential PET targets P2 × 7R, COX-2, CB_2_R and CSF-1R have been demonstrated immunohistochemically in lesions in human MS and EAE (Yiangou et al., 2006; Beaino et al., 2017; Hagan et al., 2020). Of these only P2 × 7R has been evaluated as a PET target (using the novel PET tracer [^11^C]SMW139) in MS and was reported to identify neuroinflammation in lesions and normal appearing brain tissue in patients with active relapsing remitting MS (Hagens et al., 2020). Similar to AD, microglia in MS brains exhibit a marked downregulation of microglial core genes such as P2RY12 and Tmem119 (Zrzavy et al., 2017; Masuda et al., 2019). Therefore, an area for future research is to develop PET tracers that can distinguish between homeostatic microglia and those associated with MS pathogenesis. Single cell RNA sequencing showed that brain tissue from MS patients with early active multiple sclerosis contained a mixture of microglia clusters, including three homeostatic microglia clusters and four clusters with unique disease-related molecular signatures (Masuda et al., 2019). The ability to discriminate these microglia subtypes using PET would allow for exquisite imaging of microglial dynamics in MS patients and provide new insights into disease pathogenesis. However, this remains a challenging concept as most of the genes that are enriched in MS-specific microglial clusters are also expressed by infiltrating myeloid cells and are not suitable targets for microglia-specific imaging.

### Retinal imaging biomarkers and microglia in MS

Multiple sclerosis also affects the eyes, causing thinning of the nerve fiber layer (NFL), ganglion cell layer (GCL), inner plexiform layer (IPL) and inner nuclear layer (INL) of the retina, reduced macular volume and optic neuritis (Cennamo et al., 2016; Petzold et al., 2017; Pearson et al., 2022; Usta and Gunay, 2023; Vujosevic et al., 2023) (summarized in Figure 1). Therefore, retinal imaging biomarkers are a growing area of interest for early detection and monitoring of MS. Spectral domain OCT has emerged as a valuable tool for investigating neurodegeneration in the retina and has demonstrated that increased thinning of the inner retinal layers is associated with worsening long-term disability in MS (Lambe et al., 2021; Bsteh et al., 2023; Gernert et al., 2023). OCT studies have also revealed that atrophy of the retinal NFL and GCL reflect brain atrophy in MS patients, particularly grey matter loss (Saidha et al., 2015; Cagol et al., 2023).

MS-associated ocular changes are most prominent in the inner retina; however, a recent AO-OCT study demonstrated that the outer retina is also affected. McIlwaine et al. (2023) reported that MS patients had a significantly lower cone outer-segment density compared to healthy controls; these authors also observed an increase in the thickness of the photoreceptor layer in MS patients who had a history of optic neuritis. Thickening of the combined outer plexiform and outer nuclear layers is also a feature in MS-associated optic neuritis, and this is thought to occur due to inflammation (Petzold et al., 2017). Another non-invasive indicator of inflammation in the retina is the presence of hyper-reflecting foci, which are increased in the retinae of MS patients compared to healthy controls (Pilotto et al., 2020; Pengo et al., 2022). These are thought to represent clusters of activated and proliferating retinal microglia and are associated with cortical pathology, suggesting that retinal microglia may be useful biomarkers in MS (Pengo et al., 2022).

Retinal microglia have not been well studied in MS. In the EAE model, retinal microglia undergo morphological changes consistent with an activated phenotype (Jin et al., 2019). Using cSLO, Cruz-Herranz et al. (2021) demonstrated that the density of retinal myeloid cells markedly increased during the acute phase of EAE and then decreased during the chronic phase. The same authors performed single cell transcriptomic profiling of retinal microglia and reported that these cells existed in a pro-inflammatory state prior to the onset of disease and then switched to a protective state in chronic EAE (Cruz-Herranz et al., 2021). Future applications of AO-SLO/AO-OCT may enable non-invasive characterization of retinal microglia in distinct tissue layers, and provide additional imaging biomarkers for MS.

## Conclusion

Microglia are involved in the pathogenesis of neurodegenerative diseases. Therefore, non-invasive brain and retinal imaging techniques to visualize microglia in living patients can be used to monitor disease progression. A major limitation of current imaging approaches is they lack specificity for microglia and cannot distinguish the unique microglial subtypes that have been identified in conditions such as AD and MS. To overcome these limitations, further research is needed to identify microglia subtype-specific imaging targets during different stages of neurodegeneration. Moreover, advances in *in vivo* imaging are essential to establish standardized approaches for diagnosing and monitoring the progression of neurological diseases.

## Author contributions

FE: Writing—original draft, Writing—review and editing. DH: Supervision, Writing—review and editing. AW: Writing—review and editing. SD: Funding acquisition, Supervision, Writing—original draft, Writing—review and editing.
